# Evaluation of Blood Droplet Volumes on the Cobas Plasma Separation Card for HCV RNA Testing in Resource‐Limited Settings

**DOI:** 10.1111/jvh.70091

**Published:** 2025-09-26

**Authors:** Huma Qureshi, Jesse A. Canchola, Ghayas Hai, Amtul Quddos Latif, Neil T. Parkin, Benjamin La Brot

**Affiliations:** ^1^ Doctor's Plaza Karachi Pakistan; ^2^ Roche Molecular Systems Pleasanton California USA; ^3^ Doctor's Laboratory Karachi Pakistan; ^4^ Jinnah Postgraduate Medical Centre Karachi Pakistan; ^5^ Data First Consulting, Inc. Sebastopol California USA

## Abstract

Detection of viral RNA is essential for hepatitis C virus (HCV) diagnosis. Collection and preservation of plasma, the preferred specimen type, is challenging in some areas. The Cobas Plasma Separation Card (PSC) is an alternative specimen type with no cold chain requirements. The PSC is designed to use capillary blood from fingerstick and capillary tube collection, but alternative sample collection options would broaden PSC utility. This study explored qualitative and quantitative HCV RNA detection with PSC prepared using a syringe needle, compared to plasma. Using a 24‐gauge syringe, blood was drawn by venipuncture from HCV antibody‐positive clinic patients aged > 18 years and used to prepare plasma or spotted directly onto three PSCs using 6, 8 and 10 drops per spot (group 1) or 8, 10 and 12 drops (group 2). HCV RNA was measured using the Cobas HCV assay. Test results for all conditions were available for 143 patients in group 1 and 109 patients in group 2. The proportions with detectable HCV RNA were not significantly different from plasma, and overall agreement was over 88% for any PSC spot number (Fisher exact test *p* > 0.1). The mean HCV viral load was lower for PSC samples vs. plasma for six or eight spots in group 1 but not statistically different for 10 or 12 spots in either group. Direct spotting of blood using a syringe is a viable alternative to finger prick and capillary tube transfer for PSC preparation. This approach may be beneficial in resource‐limited settings and in patient populations for whom capillary blood collection is challenging.

## 1 Introduction

Detection and measurement of hepatitis C virus (HCV) viral RNA is essential for the diagnosis and management of HCV infection [1, 2]. Specifically, an RNA test is required to confirm a current, active infection in antibody‐positive individuals and to monitor a patient's response to antiviral therapy to verify a cure.

Collection and preservation of plasma, the preferred specimen type for HCV RNA testing, can present challenges in remote and resource‐limited areas. Trained phlebotomists and equipment may not always be available, and requirements for preservation of plasma (refrigerated or frozen) cannot be met in all locations. Alternatives to plasma as a specimen type include dried blood spots (DBS) [3, 4] and the Cobas Plasma Separation Card (PSC) [5, 6, 7]. DBS are widely available and can support specimen collection for a variety of applications including RNA and DNA detection and quantification for HCV as well as hepatitis B virus (HBV) [3] and human immunodeficiency virus (HIV) [8, 9]. DBS contain cellular components (red and white blood cells and platelets) in addition to plasma, which can lead to discrepancies in nucleic acid test results compared to liquid plasma. The PSC is an alternative matrix that can be used to generate dried plasma spots for PCR analysis with no cold chain requirements, facilitating sample collection in remote, underserved areas. The advantages and limitations of DBS and PSC for nucleic acid testing for HBV, HCV and HIV, including their potential to increase access to diagnostic testing, have been reviewed recently elsewhere [10].

The PSC is designed to use capillary blood (140 μL) from fingerstick and capillary tube collection, but alternative sample collection options would broaden PSC utility. This study explored qualitative and quantitative HCV RNA detection with PSC spotted with different venous blood volumes using a syringe needle, compared with results from plasma.

## 2 Methods

This prospective non‐interventional study enrolled patients > 18 years of age who were positive for anti‐HCV antibodies attending a liver outpatient clinic in Karachi, Pakistan, from April to September 2024. This private clinic is a partner to the provincial health department for the elimination of hepatitis from the country and follows the national HCV testing and treatment guidelines. Patients were selected at random and included treatment‐naïve cases (not treated for HCV viraemia), those on treatment with direct‐acting antivirals or those who had come for verification of sustained viral response. Seriously ill patients were excluded. Informed consent was obtained according to local regulations, and the protocol was approved by the national bioethics committee.

Using a 24‐gauge needle, 5 mL of whole blood was drawn by venipuncture from consenting patients; 3 mL was transferred into a plasma preparation tube for generating plasma, which was then stored at −80°C. Initially, the remainder was spotted directly from the needle onto three PSCs (Roche Molecular Systems, Pleasanton, CA) using 6, 8 and 10 drops per spot (group 1); after an interim analysis indicated that results using six drops were suboptimal, PSCs were prepared using 8, 10 and 12 drops instead (group 2). Previous calibration studies determined that 12 drops from a 24‐gauge syringe corresponded to 140 μL. PSCs were air‐dried and stored in polythene bags with desiccant at room temperature for 10–15 days. PSCs and plasma samples were processed in a local laboratory for the measurement of HCV RNA viral load (Cobas HCV assay with the Cobas 5800 system, Roche Molecular Systems) according to the manufacturer's instructions.

Statistical analysis was performed using SAS (The SAS Institute, Cary, NC) and Prism 10 (GraphPad, Boston, MA).

## 3 Results

A total of 294 patients were enrolled between April and September 2024. Mean age was 45 years (range 18–88) and 154 (52%) were male. For 278 patients, valid HCV RNA results were available from plasma or one of the PSC sets: 166 patients in group 1 and 112 patients in group 2. Valid results for plasma and all three PSC sets were available for 255 patients: 146 patients in group 1 and 109 patients in group 2. Results could not be generated for 23 participants because of an assay reagent lot quality issue that led to invalid results, and there was insufficient specimen quantity for retesting once the issue was resolved (see Table S1).

The percentage of samples with detectable HCV RNA in group 1 was 55% in plasma and 47%, 46% and 49% in PSC made with 6, 8 or 10 drops, respectively. The percentage detectable in group 2 was 64% in plasma and 63%, 64% and 62% in PSC made with 8, 10 or 12 drops, respectively. The proportion with detectable HCV RNA in any PSC group was not significantly different from plasma (Fisher Exact test *p* > 0.1; Table S2).

**FIGURE 1:**
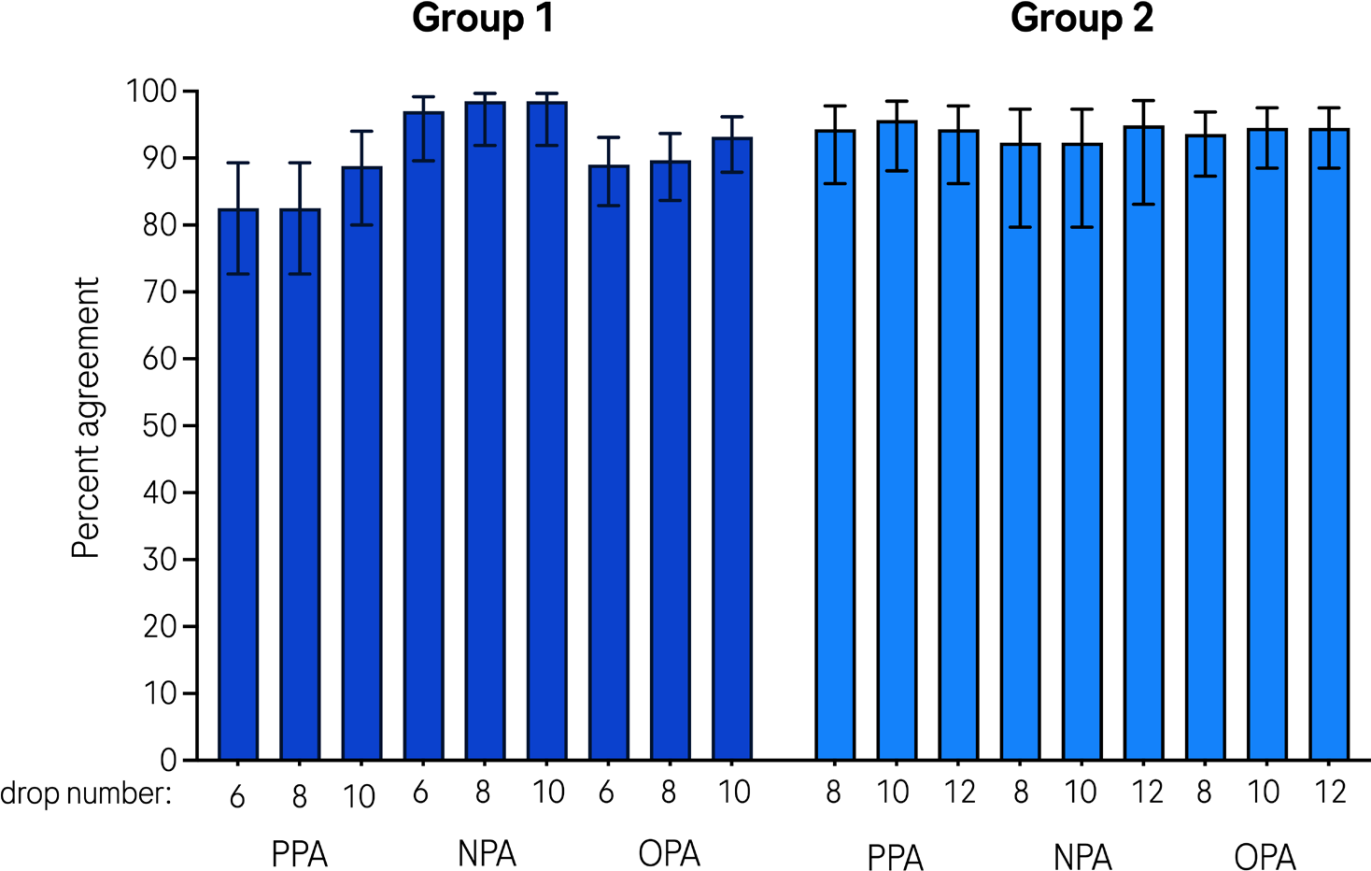
Agreement analysis. NPA, negative percent agreement; OPA, overall percent agreement; PPA, positive percent agreement.

The overall percentage agreement (OPA) for HCV detectability in PSC versus plasma was high (> 89% in group 1, > 93% in group 2) for any PSC spot number in either group (Figure 1; Table 1). In group 1, positive percentage agreement (PPA) was slightly lower (83%–89%) in PSC versus plasma, while negative percentage agreement (NPA) was over 97%. In group 2, PPA was between 94% and 96%, while NPA was between 92% and 95%. Differences between agreement levels in PSC versus plasma were not statistically significant. Because both plasma and PSC specimens were tested using the same molecular assay and no independent clinical reference standard was used, we calculated positive and negative percent agreement (PPA and NPA), rather than sensitivity and specificity. These terms reflect the degree of agreement between specimen types without implying that one specimen serves as a diagnostic gold standard. This approach aligns with current best practices for method comparison when evaluating alternative matrices using the same test platform.

**TABLE 1:** Agreement analysis.

Group	Drop no.	PSC	Plasma			Agreement measure	% (*n*/*N*)	95% CI
			Positive	Negative	Total			
1	6	Positive	66	2	68	PPA	83 (66/80)	73, 89
		Negative	14	64	78	NPA	97 (64/66)	90, 99
		Total	80	66	146	OPA	89 (130/146)	83, 93
	8	Positive	66	1	67	PPA	83 (66/80)	73, 89
		Negative	14	65	79	NPA	99 (65/66)	92, 100
		Total	80	66	146	OPA	90 (131/146)	84, 94
	10	Positive	71	1	72	PPA	89 (71/80)	80, 94
		Negative	9	65	74	NPA	99 (65/66)	92, 100
		Total	80	66	146	OPA	93 (136/146)	88, 96
2	8	Positive	66	3	69	PPA	94 (66/70)	86, 98
		Negative	4	36	40	NPA	92 (36/39)	80, 97
		Total	70	39	109	OPA	94 (102/109)	87, 97
	10	Positive	67	3	70	PPA	96 (67/70)	88, 99
		Negative	3	36	39	NPA	92 (36/39)	80, 97
		Total	70	39	109	OPA	95 (103/109)	89, 98
	12	Positive	66	2	68	PPA	94 (66/70)	86, 98
		Negative	4	37	41	NPA	95 (37/39)	83, 99
		Total	70	39	109	OPA	95 (103/109)	89, 98

**FIGURE 2:**
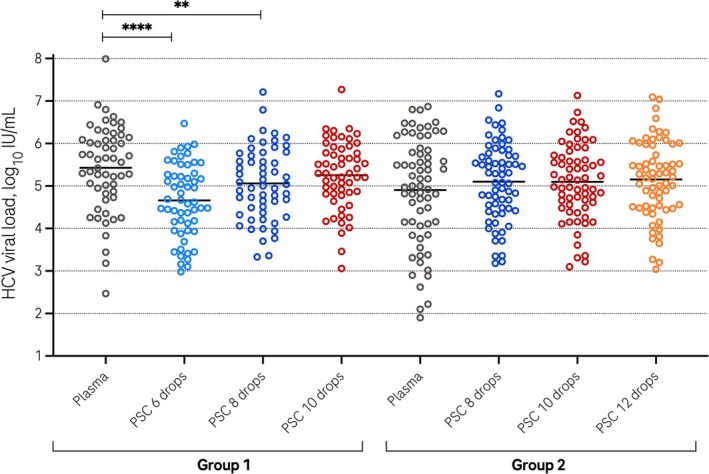
HCV viral load results. Results with HCV RNA in the linear range in plasma and all 3 PSC groups. *N* = 56 for group 1, *N* = 65 for group 2. ****Paired *t*‐test versus plasma: *P* < 0.0001; **Paired *t*‐test versus plasma: *P* = 0.007. Horizontal bars = mean.

Among samples with results in the linear range of the assay in plasma and all three sets of PSC, the mean viral load in group 1 (*N* = 56) was 5.43 log_10_IU/mL in plasma, while it was 4.66, 5.06 and 5.25 log_10_IU/mL in PSC made with 6, 8 or 10 drops, respectively (Figure 2). The differences in viral load for six or eight spots compared to plasma were statistically significant (paired *t*‐test *p* < 0.01). In group 2 (*N* = 65), the mean viral load was 4.90 log_10_IU/mL in plasma, while it was 5.10, 5.10 and 5.15 log_10_IU/mL in PSC made with 8, 10 or 12 drops, respectively (Figure 2). The differences in viral load compared to plasma were not statistically significant in group 2 (paired *t*‐test *p* > 0.05).

**FIGURE 3:**
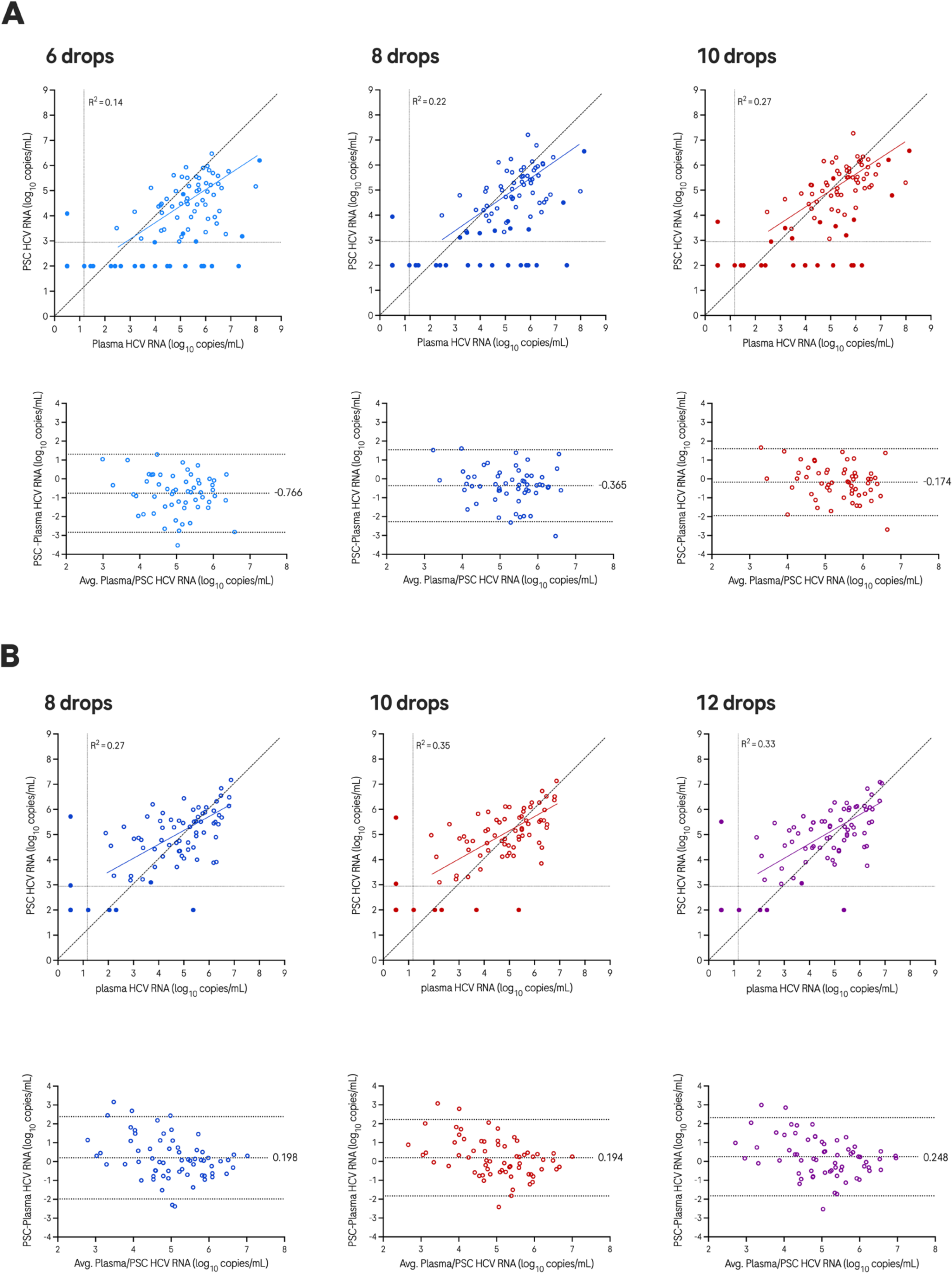
Viral load comparisons between PSC and plasma. (A) Group 1, 6, 8 or 10 drops PSC versus plasma. (B) Group 2 Results: PSCs prepared with 8, 10, or 12 drops versus plasma. Horizontal dotted line: PSC LLOQ; vertical dotted line: plasma LLOQ. Filled circles: Excluded from regression and bias calculations (one or more results outside the linear range). Top dotted line: 95% upper limit; middle dotted line: Mean bias; bottom dotted line: 95% lower limit.

Viral load measured in PSC was correlated with that in plasma (Figure 3). The coefficient of variation (*R* ^2^) versus plasma was above 0.2 for all sets except PSC made with six drops in group 1. The *R* ^2^ for 10 and 12 drops in group 2 was 0.35 and 0.33, respectively. The Deming regression slope parameter estimates were 0.7 in group 1 and 0.6 in group 2; the Y‐intercept ranged from 1.1 to 1.7 log_10_ IU/mL in group 1 and from 2.3 to 2.4 log_10_ IU/mL in group 2 (Table S3).

The mean bias estimates in viral load in group 1 PSC versus plasma were −0.8, −0.4 and −0.2 log_10_ IU/mL for 6, 8 and 10 drops in group 1, respectively. The mean bias estimates in viral load in group 2 PSC versus plasma were 0.2 IU/mL for 8, 10 and 12 drops in group 2 (Table S4).

## 4 Discussion

The use of a 24‐gauge syringe and counting drops applied to the PSC led to an equivalent proportion of participants with HCV RNA detected and mean viral load compared to plasma, as long as a minimum of 10 drops were applied per spot. Viral load measured in PSC prepared in this manner was well correlated with that measured in plasma. This indicates that direct spotting of venous blood from a syringe onto PSC may be a viable alternative to pipetting of venous or capillary blood.

Our study had some limitations. This was a proof‐of‐concept study to explore the potential for use of a syringe to prepare PSC instead of capillary tubes, and as such it did not include comparison to other PSC preparation methods such as fingerstick or venipuncture and pipetting. Our preliminary results indicate that the syringe method warrants further investigation. Different drop sizes arising from the use of different‐sized needles would likely require additional exploration to establish the appropriate drop numbers for optimal results. The range of HCV RNA concentrations in this study was not designed to fully assess diagnostic utility for RNA detection close to the detection limits. Finally, the study design did not include PSC prepared according to instructions for use (transfer of 140 μL blood using capillary tubes).

In conclusion, our study indicates that the preparation of PSC by direct spotting with a syringe may reduce logistical challenges with sample collection while maintaining high diagnostic accuracy, particularly in resource‐limited settings and in patient populations for whom finger prick and capillary blood collection are challenging (e.g., those with heavily calloused hands).

## Conflicts of Interest

This study was supported in part by Roche. J.A.C. and B.L.B. are employees of Roche Molecular Systems, Pleasanton, CA, USA. N.T.P. is an independent contractor supported by Roche Molecular Systems. Cobas Plasma Separation Cards used in this study were provided by Roche Molecular Systems. COBAS is a trademark of Roche. The regulatory status of the Cobas Plasma Separation card, Cobas HCV assay and Cobas 5800 system should be verified in each region, as the availability of these devices may vary by region. Not all products are approved for the specified use in all jurisdictions. The Cobas Plasma Separation Card is not approved for direct spotting of venous blood using a syringe.

## Supporting information


**Data S1:** jvh70091‐sup‐0001‐supinfo.docx.

## Data Availability

The data that support the findings of this study are available on request from the corresponding author. The data are not publicly available due to privacy or ethical restrictions.
