# Supplementary material for: Evaluation of Blood Droplet Volumes on the Cobas Plasma Separation Card for HCV RNA Testing in Resource‐Limited Settings
Source: J Viral Hepat. 2025 Sep 26;32(11):e70091. doi: 10.1111/jvh.70091 (PMC12474196; doi:10.1111/jvh.70091)
Supplement: Supplementary file 1 — Data S1: jvh70091‐sup‐0001‐supinfo.docx. [file JVH-32-0-s001.docx]

Supplementary Material

**Table S1.** Summary of missing data. Each row represents a participant, showing whether or not a viral load result was obtained for each condition.

| Group | Plasma | PSC 6 drops | PSC 8 drops | PSC 10 drops | PSC 12 drops |
| --- | --- | --- | --- | --- | --- |
| 1 | yes | no | yes | yes |  |
| 1 | no | no | yes | yes |  |
| 1 | yes | no | yes | yes |  |
| 1 | yes | no | yes | yes |  |
| 1 | yes | no | yes | yes |  |
| 1 | no | yes | yes | yes |  |
| 1 | yes | no | yes | no |  |
| 1 | yes | yes | yes | no |  |
| 1 | no | yes | yes | yes |  |
| 1 | no | yes | yes | yes |  |
| 1 | no | yes | yes | yes |  |
| 1 | yes | yes | no | no |  |
| 1 | yes | no | no | yes |  |
| 1 | yes | yes | no | no |  |
| 1 | yes | yes | no | no |  |
| 1 | yes | yes | no | no |  |
| 1 | yes | yes | no | yes |  |
| 1 | yes | no | yes | yes |  |
| 1 | yes | no | no | yes |  |
| 1 | yes | yes | no | no |  |
| 2 | yes |  | no | yes | yes |
| 2 | yes |  | yes | no | no |
| 2 | yes |  | yes | no | no |

**Table S2.** Detectability of HCV RNA

|  | **Group 1 (N=146)** | | **Group 2 (N=109)** | |
| --- | --- | --- | --- | --- |
|  | **N (%) detectable** | **mean HCV RNA, log IU/mL^1^** | **N (%) detectable** | **mean HCV RNA, log IU/mL^1^** |
| Plasma | 80 (55%) | 5.43 | 70 (64%) | 4.90 |
| PSC 6 drops | 68 (47%) | 4.66^1^ | - | - |
| PSC 8 drops | 67 (46%) | 5.06^2^ | 69 (63%) | 5.10 |
| PSC 10 drops | 72 (49%) | 5.25 | 70 (64%) | 5.10 |
| PSC 12 drops | - | - | 68 (62%) | 5.15 |

1 Paired t-test vs plasma: P < 0.0001 2 Paired t-test vs plasma: P = 0.007

HCV RNA % detection not significantly different from plasma for any PSC spot number (Fisher Exact test P > 0.1).

**Table S3** Deming Regression parameters

| **Group** | **Drop no.** | **Intercept, log_10_ IU/mL (95% CI)** | **Slope (95% CI)** | **R-Square** |
| --- | --- | --- | --- | --- |
| 1 | 6 | 1.1 (-1.4 - 3.6) | 0.7 (0.2-1.1) | 0.14 |
|  | 8 | 1.3 (-1.0 - 3.6) | 0.7 (0.3-1.1) | 0.22 |
|  | 10 | 1.7 (-0.2 - 3.7) | 0.7 (0.3-1.0) | 0.27 |
|  |  |  |  |  |
| 2 | 8 | 2.4 (1.2 - 3.6) | 0.6 (0.3-0.8) | 0.27 |
|  | 10 | 2.4 (1.3 - 3.4) | 0.6 (0.4-0.8) | 0.35 |
|  | 12 | 2.3 (1.2 - 3.4) | 0.6 (0.4-0.8) | 0.33 |

**Table S4.** Bland-Altman parameters

| **Group** | **Drop no.** | **N** | **Mean bias, log_10_ IU/ml (95% CI)** |
| --- | --- | --- | --- |
| 1 | 6 | 56 | -0.8 (-1.0, -0.5) |
|  | 8 | 56 | -0.4 (-0.6, -0.1) |
|  | 10 | 56 | -0.2 (-0.4, 0.07) |
|  |  |  |  |
| 2 | 8 | 65 | 0.2 (-0.08, 0.5) |
|  | 10 | 65 | 0.2 (-0.06, 0.5) |
|  | 12 | 65 | 0.2 (-0.01, 0.5) |
